# Standardizing a microbiome pipeline for body fluid identification from complex crime scene stains

**DOI:** 10.1128/aem.01871-24

**Published:** 2025-04-30

**Authors:** Meghna Swayambhu, Mario Gysi, Cordula Haas, Larissa Schuh, Larissa Walser, Fardin Javanmard, Tamara Flury, Sarah Ahannach, Sarah Lebeer, Eirik Hanssen, Lars Snipen, Nicholas A. Bokulich, Rolf Kümmerli, Natasha Arora

**Affiliations:** 1 Department of Forensic Genetics, Zurich Institute of Forensic Medicine, University of Zurich 27217, Zurich, Switzerland; 2 Department of Bioscience Engineering, Laboratory of Applied Microbiology and Biotechnology, University of Antwerp 692679, Antwerp, Belgium; 3 Department of Forensic Sciences, Oslo University Hospital 155272, Oslo, Norway; 4 Faculty of Chemistry, Biotechnology and Food Sciences, Norwegian University of Life Sciences 56625, As, Akershus, Norway; 5 Department of Health Sciences and Technology, ETH, Zurich, Switzerland; 6 Department of Quantitative Biomedicine, University of Zurich 726932, Zurich, Switzerland; University of Illinois Urbana-Champaign, Urbana, Illinois, USA

**Keywords:** microbiome, sexually shared microbiome, forensic science, machine learning, body fluid identification, standardization, OTUs, ASVs

## Abstract

Recent advances in next-generation sequencing have opened up new possibilities for applying the human microbiome in various fields, including forensics. Researchers have capitalized on the site-specific microbial communities found in different parts of the body to identify body fluids from biological evidence. Despite promising results, microbiome-based methods have not been integrated into forensic practice due to the lack of standardized protocols and systematic testing of methods on forensically relevant samples. Our study addresses critical decisions in establishing these protocols, focusing on bioinformatics choices and the use of machine learning to present microbiome results in case reports for forensically relevant and challenging samples. In our study, we propose using operational taxonomic units (OTUs) for read data processing and generating heterogeneous training data sets for training a random forest classifier. We incorporated six forensically relevant classes: saliva, semen, skin from hand, penile skin, urine, and vaginal/menstrual fluid, and our classifier achieved a high weighted average F1 score of 0.89. Systematic testing on mock forensic samples, including mixed-source samples and underwear, revealed reliable detection of at least one component of the mixture and the identification of vaginal fluid from underwear substrates. Additionally, when investigating the sexually shared microbiome (sexome) of heterosexual couples, our classifier could potentially infer the nature of sexual activity. We therefore highlight the value of the sexome for assessing the nature of sexual activities in forensic investigations while delineating areas that warrant further research.

**IMPORTANCE:**

Microbiome-based analyses combined with machine learning offer potential avenues for use in forensic science and other applied fields, yet standardized protocols remain lacking. Moreover, machine learning classifiers have shown promise for predicting body sites in forensics, but they have not been systematically evaluated on complex mixed-source samples. Our study addresses key decisions for establishing standardized protocols and, to our knowledge, is the first to report classification results from uncontrolled mixed-source samples, including sexome (sexually shared microbiome) samples. In our study, we explore both the strengths and limitations of classifying the mixed-source samples while also providing options for tackling the limitations.

## INTRODUCTION

Advances in next-generation sequencing technologies have prompted the investigation of the human microbiome for various applications including healthcare, diagnostics, and forensics (1–3). As more sequencing data is generated from different body fluids and tissues, several key insights have emerged. Among these, bacterial communities have been observed to be body-site specific and bacterial strains to be individual specific (4, 5). These findings open new avenues for the forensic investigation of biological evidence found at crime scenes, including body fluid identification (BFI) and human identification (ID) (1, 6–9). Body fluid identification from stains provides valuable information to reconstruct criminal events. For instance, in cases of sexual assault, establishing the presence of vaginal fluid or semen can have a significant impact on the investigation.

Recent research has explored the potential of bacterial community sequence data for body fluid identification (1, 6, 10, 11). This approach typically targets specific regions of the prokaryotic 16S rRNA gene to describe microbial communities, making it both cost-effective and ideal for forensics, where biological stains are often degraded. Studies have concentrated on forensically relevant body fluids and tissues, including vaginal fluid, semen, and urine, among others. These studies demonstrate that informative, body site-specific markers can be obtained, even when samples were deposited on various substrates or exposed to environmental conditions over an extended period (1, 6, 10, 11). Additionally, these forensic studies investigated the presentation of sequencing results as prediction probabilities obtained through various machine learning algorithms like tree-based (e.g., random forests or XG boost) and neural networks, among others (1, 6, 10). Despite these encouraging results, microbiome-based analyses are not yet used in routine forensic analysis due to several limitations. These include the lack of standardized laboratory and bioinformatics workflows, knowledge gaps in presenting the analysis output within a probabilistic framework, and the need for further standardization of machine learning output as evidence in court.

The bioinformatics protocols used in previous studies for amplicon sequencing of the 16S rRNA gene are diverse, reflecting the wealth of approaches and software available for microbiome analyses. However, this variation in turn highlights the need for standardized workflows in applied settings like forensic casework (12, 13). Amongst the different options available, two important bioinformatic decisions concern the clustering of read data (or not) and whether to combine publicly available data from different 16S rRNA gene regions in order to obtain larger reference data sets for comparative analyses and machine learning. Given the inherent biases associated with each 16S rRNA gene region, it is unclear whether a heterogeneous sequence combination introduces new biases (14–16).

Once these bioinformatic decisions are addressed, the second aspect to consider for forensic application is the inclusion of microbiome-based predictions as evidence. Forensic analyses typically employ a probabilistic framework for interpretation by the court, allowing the findings to be evaluated within the context of their evidentiary value (1, 17). Despite encouraging results from previous studies, machine learning tools are yet to be integrated into the forensic routine. This is due to gaps such as limited body fluid categories in the training sets and limited testing on forensically relevant samples. Biological evidence from crime scenes is often found in degraded conditions, deposited on various substrates, and present as mixtures of two or more body fluids, among other possibilities (18). While previous studies have tested machine learning classifiers on aged samples and samples on substrates, these samples were typically high input, unlike forensic evidence. Moreover, the performance of these classifiers on mixtures remains understudied (6, 9, 10).

As a first step in our study, we begin with addressing the bioinformatics aspect. We compared the resolution of operational taxonomic units (OTUs) clustered at 97% identity threshold versus amplicon sequencing variants (ASVs) for BFI purposes. Next, using OTUs generated with closed reference clustering, we combined data from nine different data sets, including public and novel data sets. These data sets were generated by sequencing different regions of the 16S rRNA gene (V1V3, V3V4, V4, and V4V5 regions). Subsequently, a random forest classifier was trained on this combined data set comprising six training classes, namely, saliva, semen, skin from hand, penile skin, urine, and vaginal/menstrual fluid. Our training data set included forensically relevant body fluids/tissues from both commonly studied body fluids as well as understudied ones, providing a comprehensive representation for the classifier’s training.

As a last step in our study, we tested our random forest classifier on mock forensic samples, including mixed-source samples generated in controlled conditions in the laboratory, as well as mock samples representing forensic evidence. Such evidence in sexual assault cases generally includes clothing items such as underwear from victims and swabs from the urogenital areas of victims and suspects (19–22). We included underwear samples from women and vaginal, semen, and penile skin swabs from couples for investigating the sexually shared microbiota (sexome). We collected extensive metadata for both these data sets, including details on sexual activities and personal hygiene. In summary, we propose the use of OTUs from different 16S rRNA gene regions, producing a heterogeneous machine learning training data set that enables us to obtain a classifier displaying high performance. In addition, we demonstrate the advantages of systematically testing our classifier for inferring body fluids/tissues from samples mimicking forensic casework.

## RESULTS

### Body site clustering patterns from OTUs and ASVs are highly similar

**Fig 1:**
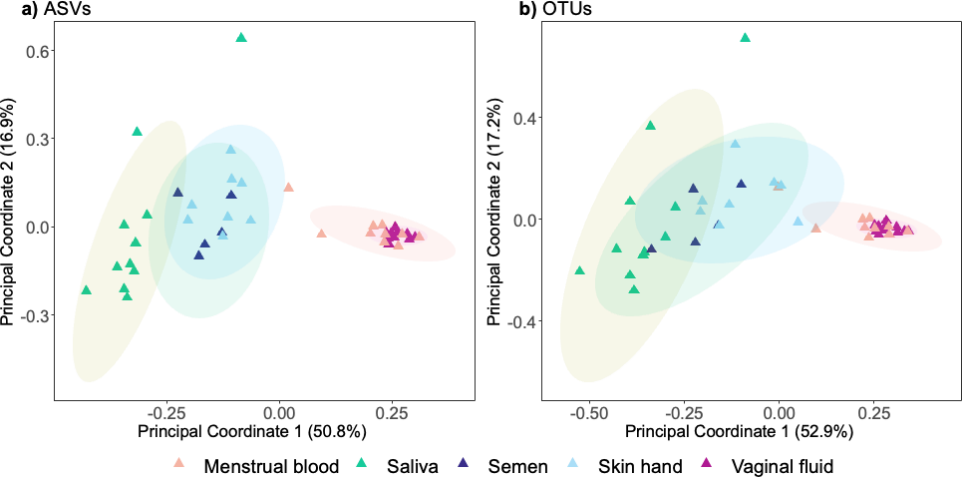
Principal coordinate analysis plots (based on the weighted unifrac distances) showing clustering of body fluid/tissue samples. (a) ASV data for samples from Dobay et al. (11) (*n* = 42, PERMANOVA F_4,41_ = 16.15, r^2^ = 0.61, *P* = 0.001). (b) OTU data clustered at 97% for samples from Dobay et al. (12) (PERMANOVA F_4,41_ = 15.95, r^2^ = 0.61, *P* = 0.001). Body fluids/tissues are color-coded*.*

We first compared the clustering patterns between closed reference OTUs (97%) and ASVs for samples from saliva, semen, skin from hand, menstrual blood, and vaginal fluid. We used a V4V5 data set of 42 samples containing 3,965 ASVs and 2,647 OTUs among the five body fluids/tissues studied. We generated PCoA plots using weighted Unifrac (UF) (Fig. 1a and b) and Bray Curtis (BC) dissimilarity distances (File S1; Fig. S1.1).

We observed that the samples clustered according to body site for both OTUs and ASV data. In all PCoA plots, irrespective of distance metric, significant effects of body fluids/tissues on clustering patterns were observed (ASV permutational multivariate analysis of variance [PERMANOVA] weighted UF, F_4,41_ = 16.15, r^2^ = 0.61, *P* = 0.001 and OTU PERMANOVA weighted UF, F_4,41_ = 15.95, r^2^ = 0.61, *P* = 0.001, ASV PERMANOVA BC F_4,41_ = 6.15, r^2^ = 0.38, *P* = 0.001 and OTU PERMANOVA F_4,41_ = 7.64, r^2^ = 0.43, *P* = 0.001). As illustrated in Fig. 1, pairwise comparisons revealed three main body fluid/tissue clusters: (i) saliva, (ii) semen together with skin from hand, and (iii) vaginal fluid with menstrual blood samples (File S2). The first two principal coordinate axes in the OTU analysis explained a slightly higher proportion of variance (70.1%) compared to the principal coordinate axes in the ASV analysis (67.7%). Regression analyses on PC1 and PC2 coordinates for both distance metrics demonstrated strong correlations between the ASV and OTU coordinates for both axes (PC1, r^2^ = 0.979 and PC2, r^2^ = 0.987) (File S1; Fig. S1.2). Overall, the clustering patterns observed for ASV and OTU data demonstrate similar distinction of body sites.

### Samples from different 16S rRNA gene regions cluster similarly

**Fig 2:**
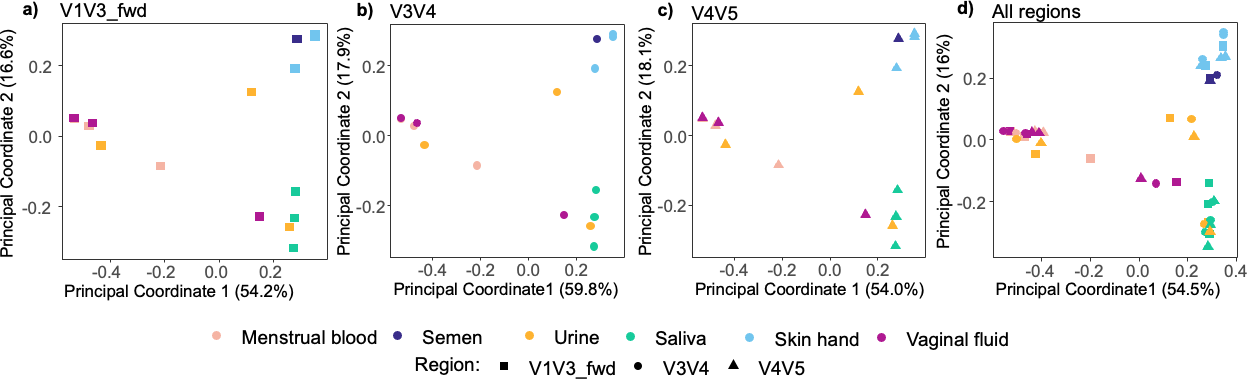
Principal coordinate analysis plots for data from the different 16S rRNA gene regions using OTUs (97%) and weighted UniFrac distances. (a) V1V3 data (*n* = 16, PERMANOVA F_5,10_ = 3.57, r^2^ = 0.64, *P* = 0.003), (b) V3V4 data (*n* = 16, PERMANOVA F_5,10_ = 4.89, r^2^ = 0.71, *P* = 0.002), (c) V4V5 data (*n* = 16, PERMANOVA F_5,10_ = 4.76, r^2^ = 0.7, *P* = 0.001), and (d) all three regions combined, V1V3, V3V4, and V4V5 (*n* = 48, PERMANOVA F_5,42_ = 15.07, r^2^ = 0.64, *P* = 0.001). Body fluids/tissues are color-coded, and the 16S rRNA gene regions are depicted by different shapes.

Next, we assessed whether OTUs for different 16S rRNA gene regions provided comparable distinction across body fluids/tissues. OTU clustering was conducted using a closed reference clustering method, a robust and computationally faster approach compared to other options like fragment insertion. We used a subset of 48 samples from the Zurich Institute of Forensic Medicine data set (herein referred to as the Zurich data set) from six different body fluids/tissues. For these samples, we had sequence data from V1V3, V3V4, and V4V5 regions (16 samples for each region), obtained using similar protocols (as detailed in File S3). Using weighted Unifrac distances, we observed highly similar clustering patterns irrespective of the gene region analyzed (Fig. 2a through c). Regression analyses between the coordinates of PC1 and PC2 of different gene regions again revealed strong correlations (R^2^-values between 0.88 and 0.98, File S1.3), confirming the highly congruent clustering. Given the highly similar clustering patterns, we agglomerated the OTU abundance tables from all 48 samples to generate one PCoA plot (Fig. 2d). As observed in Fig. 2d, the body fluid/tissue clustering pattern for the combined data set remained congruent with the patterns displayed in Fig. 2a through c. Crucially, body fluid/tissue was a significant driver of clustering patterns (PERMANOVA, *P*-values < 0.05, see Fig. 2 legend for exact *P*-values), while the 16S rRNA gene region was not (PERMANOVA F_2,45_ = 0.33, r^2^ = 0.01, *P* = 0.972). In summary, our analyses showed that the clustering of body fluids/tissues is not affected when data from different 16S rRNA gene regions is combined.

### Random forest classifier trained on a heterogeneous data set achieves high prediction accuracies

In order to generate a heterogeneous training data set, we combined read data from nine different studies. These were conducted in different laboratories with diverse protocols targeting one or more regions of the 16S rRNA gene (V1V3, V3V4, V4, and V4V5) (9, 11, 23–25). The reads originated from 457 samples from six body fluids/tissues namely, saliva (*n* = 99), semen (*n* = 57), skin from hand (*n* = 80), penile skin (*n* = 20), urine (*n* = 42), and vaginal/menstrual fluid (*n* = 159). In total, there were 6,455 OTUs across all fluids/tissues. As expected, significant effects of body site were observed (F_6,450_ = 55.33, r^2^ = 0.42, *P* = 0.001, File S1; Fig. S1.4). In addition, the 16S rRNA region and the extraction kit were also found to be significantly associated with microbiome composition, albeit with lower effect sizes (region: F_4,452_ = 6.82, r^2^ = 0.06, *P*-value = 0.001, extraction kit: F_8,448_ = 7.42, r^2^ = 0.117, *P* = 0.001, File S1; Fig. S1.4).

**Fig 3:**
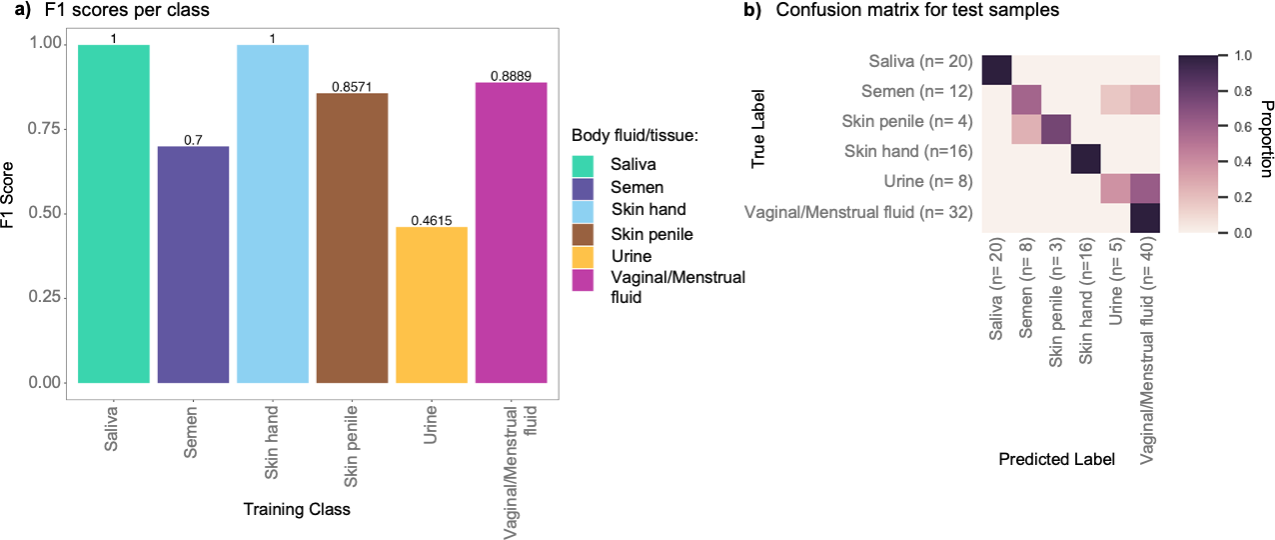
Classifier performance of the classifier trained on 365 samples on 92 test samples. (a) Barplots depicting F1 scores per class. (b) Confusion matrix for the 92 samples with sample sizes per class, the exact prediction probabilities are reported in File S4.

In the next step, we trained a random forest classifier on the OTU compositional data of six classes namely, saliva, semen, skin from hand, penile skin, urine, and vaginal/menstrual fluid. The training was done using an 80–20 train-test split, resulting in a training data set of 365 samples and a testing data set of 92 samples. Using recursive feature elimination, 281 OTUs out of 6,455 OTUs reached overall accuracies >80% (File S1; Fig. S1.5). These OTUs were then used for model training. Subsequently, we applied the trained model to the 92 test samples and assessed the reliability of the classifications using F1 scores as the performance metric. Both weighted average F1 scores and F1 scores per class were analyzed. A high weighted average F1 score of 0.89 was obtained across the six training classes (saliva, semen, skin from hand, penile skin, urine, and vaginal/menstrual fluid). The F1 scores per class varied whereby higher F1 scores were observed for saliva, semen, skin from hand, penile skin, and vaginal/menstrual fluid (0.85–1) than for semen and urine (0.70 and 0.46, respectively) (Fig. 3a). Although F1 scores provide a good overview of classifier performance, assessing individual prediction probabilities for each sample and all training classes is critical in applications like forensics. In most studies conducted so far, the accuracy of a classifier is assessed based on the highest prediction probability, whatever the value, and without setting a threshold. With this approach, 88% of the 92 test samples were correctly predicted, and 12% were categorized as misclassified. As illustrated in Fig. 3b, among the misclassifications, three semen samples were incorrectly predicted as vaginal, with varying probabilities ranging from 0.33 to 0.89. The remaining misclassifications mostly occurred amongst fluids originating from the same or proximally located sites like semen, penile skin, and urine.

Despite these overall encouraging results, some samples exhibited low probability values for the predicted class (below 0.5) or displayed similar probabilities for more than one training class. For instance, sample *A007*, which was correctly predicted as semen with a probability value of 0.38, exhibited a similarly low probability value of 0.34 for urine (data in File S4).

As an alternative approach to basing prediction solely on the class with the highest probability, we tested inferring body fluids/tissues based on probability thresholds. If the maximum probability fell below the threshold, the sample was left unclassified. Given the high microbial biomass observed in saliva, skin from hand, vaginal/menstrual fluid, and penile skin and based on the distribution of prediction probabilities, a threshold of 0.7 was chosen. A lower threshold of 0.5 was selected for fluids/tissues known to exhibit lower microbial biomass, such as semen and urine. The application of thresholds resulted in a reduction of both the proportion of correctly classified (from 88% to 77%) and misclassified (from 12% to 6%) samples. Simultaneously, it introduced a new category for unclassified samples (17%), which enabled us to acknowledge the uncertainty in predictions, especially for low bacterial biomass samples and samples originating from proximal body sites, e.g., semen, penile skin, or urine. In summary, a high number of correct predictions were obtained in most cases with or without thresholding, and most misclassifications concerned proximally located sites.

### High prediction probabilities obtained for blind single-source samples and controlled mixtures

**Fig 4:**
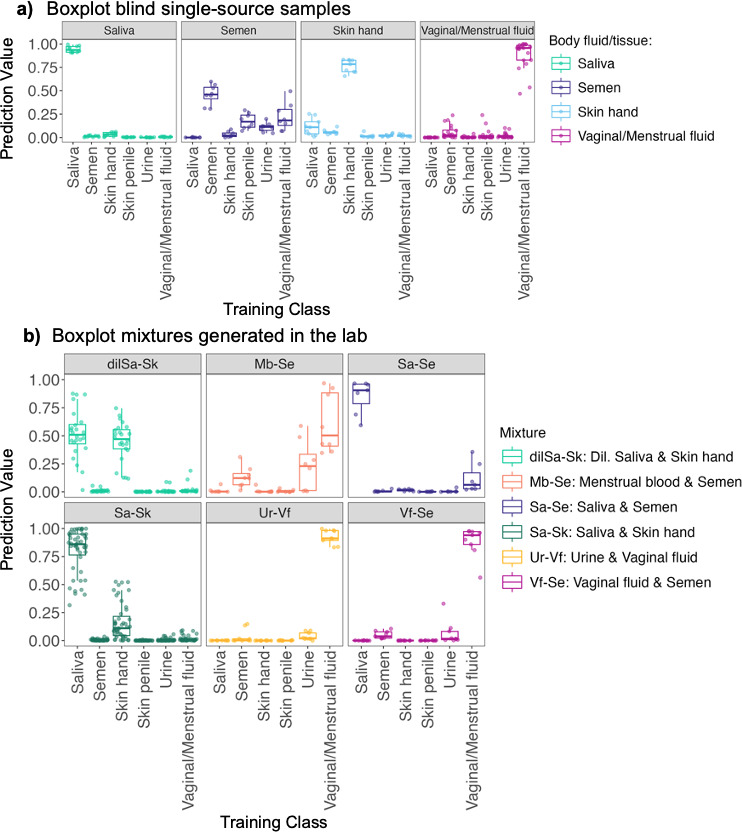
Boxplots depicting prediction probabilities of blind single-source control and mixed-source samples generated in the laboratory. (a) Blind single-source samples were faceted according to the body site. (b) Mixed-source samples generated in the lab faceted according to mixture composition. Each data point refers to a sample, and each sample has a probability value per class. Dil. Saliva & Skin refers to 1:10 diluted saliva deposited on the skin.

Once we were satisfied with the classifier performance on the test set, we used the same parameters to train an extended classifier using all 457 samples. A total of 337 OTUs from the total of 6,455 OTUs were selected for model training using recursive feature elimination (File S1, Fig. S1.6). To test this new classifier, we used independent blind controls from the Zurich data set consisting of 47 single-source samples. These comprised 10 saliva samples, nine skin (hand/forearm) samples, eight semen samples, and 20 vaginal/menstrual fluid samples. The random forest classifier performed well, yielding high F1-scores for all categories (0.85–1.00) (File S1, Fig. S1.7). The probabilities for each sample in the blind data set were visualized using boxplots (Fig. 4a). Overall, 95% and 82% of the 47 samples were correctly classified without and with thresholds, respectively (File S5). With thresholds, saliva, skin from hands, and vaginal/menstrual fluid samples were generally correctly classified (80%–100%). However, only 37% of the semen samples were classified correctly. As observed previously, unclassified samples predominantly comprised those with low microbial biomass and from proximal sites. Despite the unclassified samples, no samples were misclassified, showing that the classifier exhibited accurate performance upon assessment with an independent data set.

Next, we tested the classifier for its ability to identify body fluids from mixed-source samples since such samples are commonly encountered in forensic casework. This set comprised 104 mixed-source samples generated in the lab by mixing two sources/body fluids. For these samples, the distribution of probabilities was explored (Fig. 4b; File S6). The outcomes for this analysis were distinguished as follows: (i) where both the mixture constituents display the top two highest probabilities, (ii) where one of the mixture constituents displays the highest probability, and (iii) where neither of the two mixture constituents displays the highest probability. We observed that in 81.7% of the 104 samples, both constituents had the highest prediction probabilities. These cases comprised a large number of the saliva and skin samples (71 out of 104 samples), in line with the observation that saliva and skin are comparable in microbial load and biomass, unlike other fluids/tissues such as menstrual blood and urine. Among the remaining mixtures, and with the exception of two samples (16.3% of the 104 samples), at least one of the components had the highest prediction probability. For menstrual blood-semen, saliva-semen, urine-vaginal fluid, and vaginal fluid-semen, the component that was generally identified was the one with higher microbial load, namely vaginal/menstrual fluid or saliva. Lastly, in 1.9% of the samples, neither of the two mixture constituents displayed the highest prediction probability. These samples consisted of menstrual-semen mixtures and exhibited the highest probability for the class urine. To determine whether there were significant differences among the means of the different classes, an analysis of variance with Tukey post hoc analysis was conducted, corroborating the interpretations of our findings (File S7). In summary, we observed that in most cases, the classifier could reliably identify at least one of the mixture constituents, especially the ones that are high in microbial load.

### The classifier can detect expected traces in underwear and sexome samples accurately

In order to assess the suitability of the classifier in forensic casework, the classifier was tested on non-controlled mock samples, mimicking the samples collected during forensic casework. The predicted class was determined to be the body site with the highest prediction probability.

**Fig 5:**
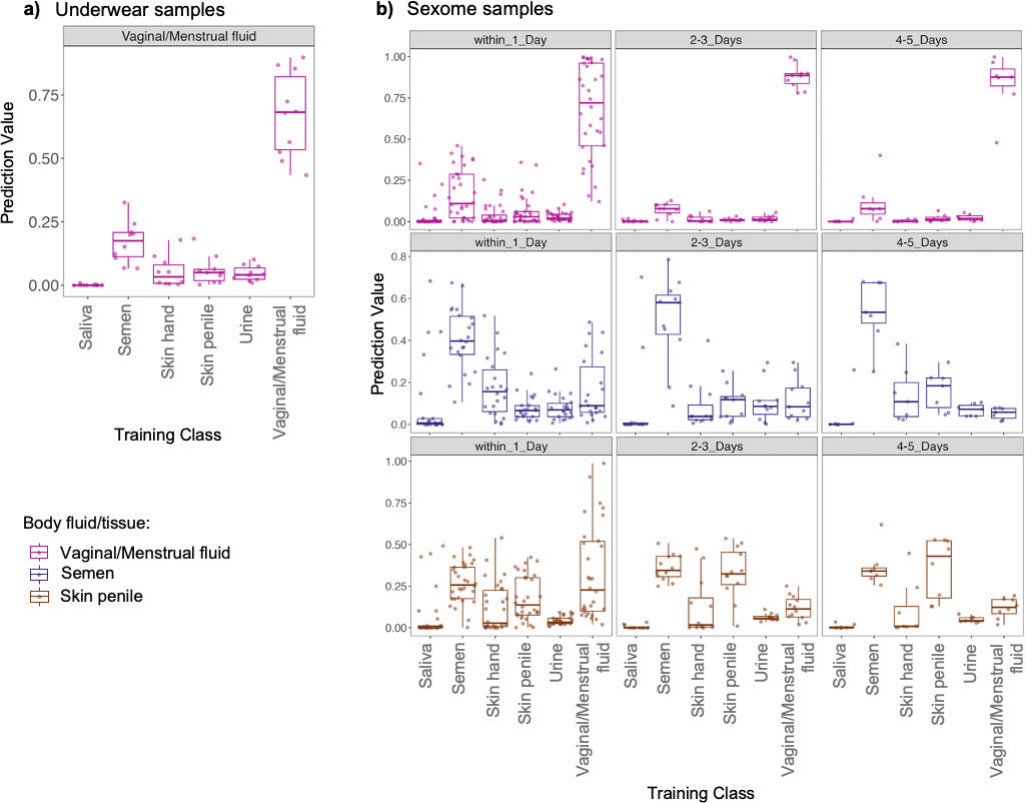
Boxplots displaying prediction probabilities for inferring urogenital microbiota from underwear and sexome samples. (a) Underwear samples. (b) Sexome samples based on days elapsed between sexual activity and sample collection. Each data point refers to a sample, and each sample has a probability value per class.

The first set of non-controlled mock samples consisted of underwear samples collected from 10 women after wearing the garment for 24 h. Prediction probabilities for all 10 underwear samples are displayed as boxplots (Fig. 5a). All samples were predicted as vaginal/menstrual fluid, and the prediction probabilities ranged between 0.43 and 0.89. We observed some signatures for semen and expected minor signatures for skin from hand (File S6). In sum, the classifier could detect microbial taxa for the vaginal body site from underwear as the substrate.

**Fig 6:**
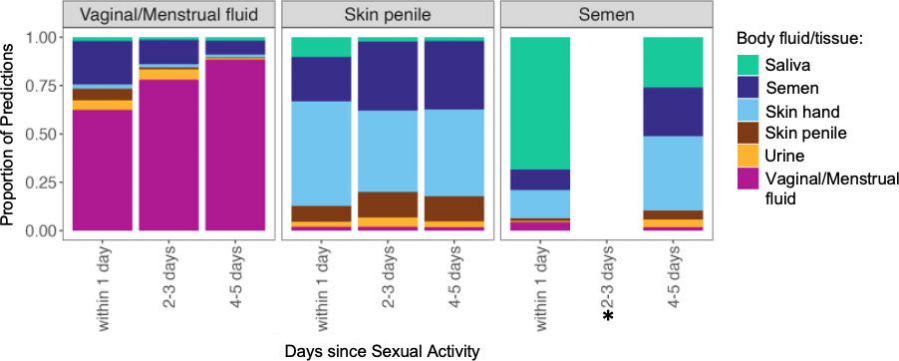
Sexually shared microbiota (vaginal/menstrual fluid, penile skin, and semen) from couple 21. For this couple, based on the questionnaires, oral, semen, and penile traces were expected in the samples collected. The semen sample for the time category 2–3 days is not available as it was excluded through quality filtering (less than 1,000 reads).

The second set of non-controlled mock samples was obtained as part of the Zurich study of urogenital samples from 22 heterosexual couples. The goal of the study was to investigate sexually shared microbiota (sexome) and to establish whether sexual activity can be inferred through the detection of mixtures of body fluids/tissues. Such a possibility would be useful for the analyses of sexual assault cases, where detecting both male components, such as penile skin or semen, and female components from the vaginal area on collected evidence would be beneficial. The data set consisted of a total of 139 samples collected/deposited on swabs including vaginal fluid, semen, and penile skin. For all these samples, metadata on sexual activities and personal hygiene was also collected. Across all days, and without applying any thresholds for the predictions, we observed 89% of vaginal fluid samples, 67% of semen samples, and 28% of penile skin swabs predicted as the respective class (File S6). Subsequently, we investigated the prediction probabilities after categorizing the data into three groups, based on the days since sexual activity: 1 day, 2–3 days, and 4–5 days. As illustrated in Fig. 5b, prediction probabilities indicated a mixing of the male and female components, especially in samples collected within 1 day after sexual activity. However, these probabilities become less indicative of such mixing as more days pass since sexual activity. Nonetheless, in one vaginal sample (V2t1), semen traces and in one penile skin sample (P16t2) vaginal traces could be detected 4 days after sexual intercourse (Files S6 and S8). In addition, the prediction probabilities observed are also indicative of the nature of sexual interactions. For samples where oral traces were expected (as specified in the metadata), saliva could be detected up to 4 days after sexual activity. As an example, in the vaginal/menstrual fluid, semen, and penile skin samples from couple 21, microbial signatures indicative of saliva in addition to skin and vaginal fluid could be detected (Fig. 6). Interestingly, the prediction probabilities indicative of oral or sexual intercourse (for instance saliva or vaginal fluid on penile skin) were lower on the fourth day after sexual intercourse. In summary, the ability to detect the intermixing of traces and nature of sexual interactions could provide important insights in forensic cases.

### Analysis of informative taxa provides explanations for the predictions

**Fig 7:**
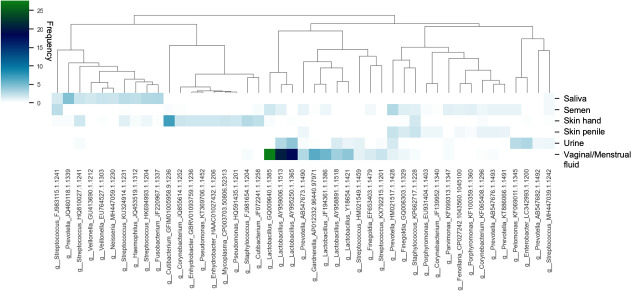
Heatmap of 10 most informative OTUs for each training class with genus level taxonomy. Forty-five out of the 337 OTUs are displayed.

Finally, we evaluated the informative taxa using feature importance scores that can be extracted from random forest classifiers (RFCs) to identify and rank the features that contributed most to the model predictions. The top 10 informative taxa per training class were selected, resulting in a total of 45 out of 337 OTUs. As shown in Fig. 7, the most informative taxa used to classify saliva, skin from hand, penile skin, and vaginal and menstrual fluid consist of distinct and characteristic OTUs, explaining the high prediction probabilities obtained for the correct classes in our study. For instance, among the taxa with the highest feature scores in saliva, we find *Veillonella* and *Fusobacterium,* whereas skin from hand was characterized by OTUs from *Cutibacterium*, *Pseudomonas*, *Staphylococcus*, *Corynebacterium,* and *Enhydrobacter*. In the case of urine, among the top 10 informative taxa, we find two OTUs, *Pelomonas* and *Enterobacter*, that are characteristic of this body site as well as other OTUs also found at other body sites. Overlapping OTUs that hinder the distinction of body sites are generally found for body fluids/tissues that are proximally located such as urine (from females) and vaginal/menstrual fluid. Nonetheless, some characteristic OTUs, like *Enterobacter* and *Prevotella,* were found in urine. In addition, the differences in relative abundance of *Lactobacillus, Gardnerella, and Streptococcus* led to a distinction of vaginal/menstrual fluid and urine. Similarly, the most informative markers for penile skin and semen overlap, making the distinction of these proximally located sites challenging. Nonetheless, the difference in frequencies of some of these shared OTUs, such as *Prevotella*, *Porphyromonas,* and *Corynebacterium,* among others, resulted in the overall distinction between these two classes. We observed overlapping taxa with similar frequencies in some classes, providing explanations for our misclassifications among semen, penile skin, and urine in males and urine and vaginal/menstrual fluid in females. For instance, vaginal/menstrual fluid samples were enriched with *Lactobacillus*, *Streptococcus,* and *Gardnerella*; however, these OTUs were also observed in low frequencies in semen or urine samples. Similarly, overlapping *Lactobacillus* and *Pelomonas* OTUs were seen between semen and urine classes. Therefore, the investigation of the top informative taxa provides possible explanations for the findings in our study.

## DISCUSSION

In our study, we addressed two key aspects for the standardization of protocols for forensic utility, namely, the selection of bioinformatics and the inclusion of microbiome-based predictions as evidence for court. Regarding the bioinformatics aspect, previous comparisons of OTUs and ASVs have demonstrated that ASVs may enable a more precise characterization of microbial diversity, as well as improved detection of rare taxa (2, 15, 26–29). At the same time, ASVs have been shown to cause errors in relative abundance calculations by providing inflated numbers of ASVs and resulting in genome splitting (30). Overall, it appears that the advantages and disadvantages of ASVs and OTUs depend on the goal. The results of our study highlight that both OTUs and ASVs from targeted 16S rRNA gene regions offer comparable resolution for forensic BFI. In studies focusing on goals like biomarker discovery or detection of (rare) bacterial strains, even deeper resolution obtained through long read or shotgun sequencing may be needed (7, 31, 32). However, the comprehensive identification of diversity, as well as rare taxa, is not needed for forensic body fluid identification. In this study, we demonstrate that ASVs allow for the distinction of body sites, but the comparison of studies based on ASVs is more challenging. Importantly, OTU clustering opens avenues to harness the combined power for different 16S rRNA gene regions.

Our analyses on the integration of data from different 16S rRNA gene regions showed that sample clustering patterns were driven primarily by the body site rather than the region. These results are congruent with those of other studies investigating the potential of combining data from different 16S rRNA gene regions (15, 16). For example, Jones et al. (15) demonstrated that combining data from different 16S rRNA gene regions offers a higher taxonomic resolution and a more comprehensive view of bacterial diversity compared to using single regions, each of which is susceptible to biases. Our classifier, trained on the combined data set, effectively identifies informative taxa for body fluid classification across the four regions incorporated in this study (V1V3, V3V4, V4, and V4V5). Additionally, Tackmann et al. (16) showed that machine learning classifiers trained on large, combined data sets achieved higher accuracies than classifiers trained on smaller data sets comprising a single region of the 16S rRNA gene. Therefore, such a comprehensive data set is not only beneficial in the forensic context but also in other applications seeking to characterize the human microbiome and to predict disease states (33).

The use of machine learning algorithms leads us to the next forensic aspect addressed in this study: the inclusion of microbiome-based predictions as evidence for court. This aspect encompasses obtaining reliable prediction probabilities from forensically relevant samples and the presentation of these probabilities in an evaluative report. As the first step to obtain prediction probabilities, we trained an RFC. Our evaluation of F1 scores per class for assessing the reliability of our classifications revealed that our accuracies were comparable or slightly higher than those reported in previous studies (1, 16). Once the training was complete, we evaluated the performance of our classifier through F1 scores (per class) (Fig. 3a). The slight improvement in our F1-score for semen in comparison to Wohlfahrt et al. (1) could be due to the heterogeneous training data set employed here, combining various 16S rRNA gene regions instead of only the V4 region alone. Another potential explanation for our findings could be the use of slightly varied training classes in both studies. While our study lacked the extensive sample sizes of Tackmann et al. (16), our F1 scores were highly comparable for the overlapping body fluids/tissues, namely, saliva, skin from hand, and vaginal. In our study, we additionally included other understudied forensically relevant samples like semen, penile skin, and urine, albeit with smaller sample sizes. We observed variations in per-class F1-scores, which could be due to technical factors like sample sizes or biological factors such as microbial diversity, biomass, or physiological sample location. These factors may also explain the lower prediction probabilities observed for urogenital samples when compared to saliva, skin, and vaginal/menstrual fluid. Analysis of the blind single-source samples using the classifier trained on the entire data set of 457 samples yielded overall higher F1-scores compared to the test set (File S1, Fig. 1.7). The increased per-class F1-scores could potentially indicate the significant impact of the larger sample sizes on classifier performance.

In evaluating misclassifications to assess the reliability of our classifications, we found that our classifier encountered difficulties in accurately classifying certain body fluids, particularly semen samples. Potential explanations for these challenges include low microbial diversity and biomass, and contamination from proximate sites. Another important explanation for the vaginal/menstrual fluid false positives in some semen samples is the absence of metadata regarding the sexual activities of these donors. Thus, it remains unclear whether these samples were genuinely single-source at the time of collection; samples with the above limitations will rarely achieve high F1-scores and accuracy.

Even though F1-scores provide an insightful overview of classifier performance, our findings highlight the importance of evaluating prediction probabilities for each class and sample. An avenue to increase confidence in our correct predictions is to employ thresholds for classifying single-source samples and introduce an “unclassified” category. This approach serves to minimize both false positives and false negatives, a critical consideration in applied settings (34, 35). While Diez-Lopez et al. (10) previously adopted a constant arbitrary threshold of 0.7 across all body fluids/tissues, we adopted flexible thresholds due to the varied microbial biomass of our training classes. A threshold of 0.5 was used for semen and urine samples, and a threshold of 0.7 was applied to other single-source samples. Of the five semen samples in the test set, two were biological replicates, with one sequenced at the V3V4 region and the other at the V4V5 region. Interestingly, upon applying thresholds, the sample sequenced with the V4V5 region fell into the unclassified category, whereas the V3V4 sample remained misclassified. One possible explanation could be the lower number of semen samples sequenced at the V3V4 region compared to the number of semen samples sequenced at the V4V5 region in the training data set.

Although thresholding showed promise in single-source samples, such an approach is not possible with mixed source samples. Our findings from the controlled mock mixtures generated in the lab showed that disentangling both constituents of all mixtures was challenging and prompted further considerations. A worthwhile strategy is to implement thresholds for the cumulative probabilities associated with the relevant body fluids and tissues (Fig. 6). However, a forensic scientist must not be privy to information on relevant fluids/tissues during evaluation. Therefore, when establishing a standardized workflow, it may be beneficial to include a predefined set of relevant body fluids for specific case types, such as sexual assault cases. Such an approach can then be consistently applied across all sexual assault cases, irrespective of the specific details of any particular case, ensuring a robust and uniform framework. In addition, our analyses also highlight the importance of setting a lower threshold to determine “undetected” body fluids/tissues among the training classes. Given these complexities, other advanced tools like multi-class classifiers or Bayesian Networks offer a promising avenue for forensic use. Bayesian Networks have been previously used for forensic DNA short-tandem repeat analysis and could potentially consolidate all probability distributions obtained for a sample and provide a resultant likelihood ratio (36).

Our analyses from the underwear samples and sexome samples showed that the classifier can detect signatures from substrate samples and that samples from couples could indicate the time frame and nature of sexual activity. Underwear has been previously studied in the context of vaginal health; however, such samples could potentially offer another avenue for detecting sexual activities (37, 38). However, our study was not specifically designed to explore this aspect, and further investigations on the underwear microbiome are necessary to assess its forensic applicability. The findings from the sexome samples yielded further interesting insights for forensic utility. Our findings were consistent with a case report focused on health-related aspects of the microbiome where microbial taxa indicative of sexual activity could be detected in vaginal and penile skin samples for up to 4 days (39). In addition, our investigation uncovered the presence of oral traces also detectable up to 4 days after sexual intercourse, offering additional understanding of the nature of sexual activities. Knowledge of these time frames coupled with analyzing the proportional decrease in male and female components therefore provides potential for establishing timelines of sexual activities in case work. Our results demonstrate that the sexome can provide additional lines of evidence or strengthen the evidentiary value of other traces, as suggested by our findings and those of Dixon et al. (40). Despite their limited sample size of 6 couples in total, the authors demonstrated unique male and female bacterial signatures before intercourse and significant transfer of the female microbiome to male partners after unprotected sex. Therefore, further research including larger cohorts and pre-coital and post-coital samples from the same couples is essential to fully leverage the sexome’s potential in forensic casework. Most of the current sexome studies primarily focus on investigating vaginal health and dysbiosis (3, 41, 42), and the findings from health and forensic research offer valuable knowledge that benefits each other. In a recent study by Carter et al. (32), the strain-level identification of bacterial taxa shared between couples is shown in a health context. Such identification can also be applied to forensics for donor identification. Notably, our study represents, to the best of our knowledge, the first application of machine learning to predict sexome samples of this scale. Our findings show that the generation of larger sample cohorts studying the transmission, persistence, and recovery of the microbial signatures between sexually involved individuals is warranted. Results from such cohorts may benefit both forensic science and health-related research, such as transmission pathways of urogenital bacteria (32).

Our study highlights the strengths of microbiome-based approaches, particularly in resolving mixed-source samples and inferring the nature of sexual activity. However, there are also limitations: for instance, body fluids/tissues with low bacterial load pose challenges for accurate classification. Nonetheless, a potential future approach is to integrate various approaches for body fluid/tissue identification, leveraging other types of markers, such as mRNA, DNA methylation, protein, and metabolite markers (12, 43–45). Each of these markers has its own advantages and disadvantages. For instance, both DNA methylation markers and mRNA markers allow, in principle, the distinction of blood, vaginal secretion, saliva, and semen. However, urine and skin have either not been tested comprehensively or cannot be reliably detected (46–48). Another important issue concerning DNA methylation markers is that the amount of DNA required to make accurate inferences may not be available in forensic traces (48). Other markers such as proteins and metabolites have been tested for the distinction of saliva, blood, urine, and semen but not for other body fluids/tissues such as vaginal fluid or menstrual blood (49–52). Thus, it is clear that further in-depth studies for each of these markers are necessitated, and particularly the exploration of forensically relevant conditions, for instance, “sexome” samples including penile skin, which remain underexplored. Additionally, while the impact of aging on single-source samples has been studied (10, 11, 53, 54), little is known about how prolonged exposure affects samples derived from multiple sources. An interesting future approach to make use of the strengths of each type of marker is to develop an integrated workflow allowing for the parallel analysis of DNA, RNA, proteins, and metabolites.

### Conclusion

In our study, we highlight important decisions on two key aspects, bioinformatics and evaluative reporting, to integrate microbiome-based analyses in forensic casework. Moreover, we propose a novel RFC trained on a heterogeneous data set including forensically crucial and underexplored body fluids/tissues like semen, urine, and penile skin and systematically test it on complex samples. Our study, therefore, provides an outlook on employing machine learning in forensics along with investigating the sexome in future studies. We provide a resolution on some aspects and highlight imminent gaps that should be addressed for microbiome analyses to be incorporated in forensic body fluid identification.

## MATERIALS AND METHODS

### Description of all data sets, controls, and mock samples

In this study, we combined data from nine different microbiome data sets comprising four unpublished studies and five published studies targeting the V1V3, V3V4, V4, and V4V5 16S rRNA gene regions. Across all studies, a total of 788 samples from seven different body fluids/tissues namely saliva, semen, menstrual blood, vaginal fluid, urine, and skin from hand and penile skin were included. Figure S1.8 describes the number of samples in all categories.

### Data from published studies and the ISALA project

Read data for the V3V4 region, V4V5 region, and V4 region from single source control and mixed source samples were obtained from the studies of Hanssen et al. (9), Dobay et al. (11), and Cauwenberghs et al. (25). Furthermore, V4 region read data from the studies of Seashols-Williams et al. (24) and Meisel et al. (23) were downloaded using q2-fondue in QIIME2 (v2023.5). In addition, vaginal V4 data from the Isala project generated following the methods of Lebeer et al. (22) was obtained from the ISALA project team. The sample IDs of all samples, as well as information on SRA accession numbers for publicly available data sets, are provided in the metadata files in File S8.

### Data generation for unpublished studies: Zurich data sets

The four unpublished data sets were generated in our lab at the Zurich Institute of Forensic Medicine and are referred to as Zurich or Zurich data sets throughout the manuscript and the metadata. These data sets targeted the V1V3, V3V4, and V4V5 regions of the 16S rRNA gene. One V1V3 data set (Zurich data set 1) was generated using slightly different protocols, and the remaining V1V3, V3V4, and V4V5 samples (Zurich data set 2) were generated using the same protocols. All four data sets consisted of different numbers of control, mixed-source, and substrate samples.

### Sample collection for Zurich data sets

Participants contributing to the Zurich data sets were recruited during the course of the different studies conducted between 2020 and 2023. All participants were provided with an informed consent form under the project number (2021-11c) according to the CEBES (Checkliste für den Ethik-Begutachtungsprozess von nichtbewilligungspflichtigen empirischen Studien) guidelines. All single-source samples were collected directly on cotton swabs or pipetted onto swabs from collection tubes/containers (see details in File S3). Controlled mock mixture samples were prepared in the lab. In the non-controlled mock samples, underwear samples were collected after women had worn the garment for 24 h, and sexually shared microbiome (sexome) samples were collected and deposited on swabs. Appropriate blanks and negative controls were included. Donors for the underwear and sexome samples were asked to fill out questionnaires including details on sexual activities and personal hygiene.

### DNA extraction and quantification for Zurich data sets

DNA extraction for all samples was conducted using one of the following kits with minor modifications in the protocols: QIAamp BiOstic Bacteremia DNA Kit (Qiagen, Germany), DNeasy PowerSoil Pro Kit (Qiagen, Venlo, Netherlands), DNA purification from buccal swabs (Spin protocol) from the QIAamp DNA Mini Kit (Qiagen, Venlo, Netherlands), and PrepFiler BTA Automated Forensic DNA Extraction Kit (Thermo Fisher Scientific, Waltham, MA, USA). The swab was either directly added to the Powerbead tubes, or the cotton was separated from the wood with a sterile scalpel and placed into a 2 mL tube (Investigator Lyse & Spin Basket Kit, Qiagen, Venlo, Netherlands). The samples were lysed according to each protocol, and the lysate was separated from the swab by centrifugation. The extraction was then carried out following the manufacturer’s protocols with minor modifications (as detailed in File S3).

DNA quantification was performed using one of the following protocols: Quantus Fluorometer (Promega, Inc., Madison WI, USA) or a SYBR green assay with the FemtoTM Bacterial DNA Quantification Kit (Zymo Research, Irvine, CA, USA) or the protocol described by Seashols-Williams et al. (24) using the PerfeCTa SYBR Green SuperMix (Quantabio, Beverly, MA, USA) and the ZymoBIOMICS Microbial Community DNA Standard D6306 (Zymo Research, Irvine, CA, USA). Cycle numbers for the amplicon PCRs were decided based on the Ct numbers obtained during the qPCR (as detailed in File S3).

### Library preparation and sequencing Zurich data sets

Bacterial communities were obtained by amplifying V1V3, V3V4, and V4V5 regions of the 16S rRNA gene. The V1V3 region was amplified using modified F27/R534 primers with spacers, the V3V4 region using F341/R806, and the V4V5 region using modified F806/R926 primers. The exact primer sequences with spacers are provided in File S3. All primers were ordered from Sigma (Darmstadt, Germany) or Microsynth AG (Balgach, Switzerland). The PCR protocol and cycling conditions were optimized per region and Zurich data set and are provided in File S3. PCR products were cleaned up using the AMPure XP beads (AMPure XP, Beckman Coulter). Single-size selection was performed for the V1V3 and V3V4 regions (small fragments <400 bp were discarded) according to the section “Clean Up Libraries” in the Illumina protocol with minor modifications. A double-size selection was performed for the V4V5 region (discarded fragments >670 bp and <400 bp) following the Ampure XP bead upper & lower cut protocol of DNA Technologies Core (55) . The integrity of the fragments was checked using 1.5% agarose gel, and the protocol is provided in File S3. The Nextera XT Index Kit v2 sets A, B, and C (24 indexes, 384 samples; Illumina) were used for dual indexing. Index PCR protocol and cycling conditions were optimized for the two Zurich data sets, and details are provided in the File S3. The Index PCR product was purified using the same clean-up protocols as described above. The library was quantified using the QubitTM dsDNA HS Assay Kit (Invitrogen, Carlsbad, CA, USA) on a Qubit Flex Fluorometer, following the manufacturer’s protocols. The libraries were normalized to 4 nM (diluted with low-TE buffer), pooled, and denaturation was conducted following the Illumina 16s metagenomics guide. The final pooled library was diluted to either 6 pM, 10 pM, or 14 pM, and 5% or 10% PhiX control was used depending on the run. Paired-end reads of 2 × 300 bp or 2 × 250 bp, depending on the run, were obtained using Illumina MiSeq FGx Reagent micro kits (600-cycles), MiSeq Reagent Kit V3 (600-cycles), or Illumina MiSeq nano kits (500-cycles) on the Illumina MiSeq sequencing platform (Illumina, Inc., Hayward CA, USA) (further details are provided in File S3).

### Read data processing for all data sets

Primers were removed for the Dobay et al. (11) and the V1V3 Zurich data set 1 using cutadapt (v3.5) and using cutadapt (v4.0) for a subset of V1V3 and V4V5 Zurich data set 2. For the remaining V3V4 and V4V5 Zurich data set 2, primers were removed using the trimLeft option in DADA2 (56) (details are provided in File S3). All data sets were processed using the DADA2 pipeline (version 1.16) with data set-specific quality filtering parameters summarized in File S3. Only forward reads were used for V1V3 samples from the Zurich data set 1 due to the poor quality of reverse reads. ASV abundance tables were clustered into OTUs at 97% similarity threshold against the SILVA database (v138, 2019) for each data set separately. The SILVA database was also clustered at 97% using the RESCRIPt plugin from QIIME2 (57–60). Decontam (v1.16) was used for all data sets separately on reagent blanks with the prevalence method and threshold of 0.1 (61). OTU abundance tables from all data sets were agglomerated using OTU IDs with MASS, reshape2, dplyr, and data.table libraries in R (v4.2.1). Samples with less than 1,000 reads were removed. The phylogenetic tree was constructed using the full-length sequences of all the OTU IDs from the single-source control samples (457 samples). Full-length sequences for all OTU IDs were extracted from the same 97% clustered SILVA database that was used during closed-reference OTU clustering. A rooted phylogenetic tree was created using MAFFT and FASSTREE using QIIME2 (v2023.5). Further processing was conducted using the phyloseq package in R (v4.2.1). OTU abundances were normalized using total sum scaling, and beta diversity with Bray-Curtis and weighted Unifrac distances were explored using phyloseq, vegan, rbiom, and ggplot2 packages in R. All regression analyses were conducted in R (v4.2.1).

### Machine learning with a random forest classifier for all data sets

A total of 457 single-source control samples and 6,455 OTUs were obtained. Data for control samples consisted of sequence data from all four regions of the 16S rRNA gene and from saliva, semen, skin-penile, skin from hands, urine, and vaginal/menstrual fluid. The vaginal fluid and menstrual blood categories were collectively referred to as “vaginal/menstrual fluid” since they share the same microbial ecosystem. To obtain prediction probabilities through machine learning, we opted for an RFC based on the results of the comparative assessment of Wohlfahrt et al. (1) demonstrating its higher performance over other classifiers. The RFC was trained with an 80–20 split using the q2-sample-classifier plugin in QIIME2 (v2023.5) using --p-estimator RandomForestClassifier, --p-optimize-feature-selection, and --p-parameter-tuning options (62, 63). The final RFC was trained on a set of 281 OTUs determined by recursive feature extraction in the --p-optimize-feature-selection option, and fivefold cross-validation was conducted with --p-n-estimators 500. Receiver operating curves, the area under the curve, and confusion matrices were generated while running the q2-sample-classifier command (62, 63). Precision, recall, and F1-scores were calculated using the Metrics and caret packages in R (v4.2.1).

Furthermore, another RFC was trained on the entire data set of 457 samples and a final set of 337 OTUs using the same hyperparameters and the same QIIME2 parameters and options as before. Using this classifier, 50 blind single-source samples and 285 mock samples, including both controlled and uncontrolled mock samples, were predicted. All boxplots and stacked bar plots were generated using the ggplot2, tidyr, tibble, ggrepel, and dplyr packages in R (v4.2.1) with the assistance of ChatGPT for troubleshooting.

Informative feature analyses were done by first extracting the taxonomy for all 337 OTUs from the same SILVA database as the closed-reference clustering using the QIIME rescript filter-taxa command. The top 10 OTUs per training class (45 distinct OTUs) were obtained from the output of the sample-classifier heatmap plugin in QIIME2. A heatmap was generated containing only these OTUs.

## Data Availability

The Zurich data sets generated in this study, as well as that of Dobay et al. (11), have been deposited in NCBI under the BioProject accession number PRJNA1201188 (BioSample accession numbers SAMN45928452 to SAMN45929019). We provide the accession numbers to all other publicly available data sets used in this study in the metadata text files in File S8. The Isala project vaginal data are available under NCBI BioProject accession number PRJEB83455 (22). The data produced by Hanssen et al. (9) are available from the authors upon request.

## SUPPLEMENTAL MATERIAL

File S1 aem.01871-24-s0001.pdf Additional PCoA plots, regression analyses, performance metrics of the classifiers, and sample design for the mock data sets.

File S2 aem.01871-24-s0002.pdf Pairwise comparisons for OTU vs ASV plots.

File S3 aem.01871-24-s0003.pdf Details for laboratory and computational methods.

File S4 aem.01871-24-s0004.txt Prediction probabilities for test data set.

File S5 aem.01871-24-s0005.txt Prediction probabilities for blind data set.

File S6 aem.01871-24-s0006.csv Prediction probabilities for all mock data sets.

File S7 aem.01871-24-s0007.csv ANOVA results for lab mixtures.

File S8 aem.01871-24-s0008.txt Metadata files for training and mock data sets used in the study.

File S9 aem.01871-24-s0009.docx Legends for all supplemental material.

ASM does not own the copyrights to Supplemental Material that may be linked to, or accessed through, an article. The authors have granted ASM a non-exclusive, world-wide license to publish the Supplemental Material files. Please contact the corresponding author directly for reuse.

## References

[1] Wohlfahrt D, Tan-Torres AL, Green R, Brim K, Bradley N, Brand A, Abshier E, Nogales F, Babcock K, Brooks JP, Seashols-Williams S, Singh B. 2023. A bacterial signature-based method for the identification of seven forensically relevant human body fluids. Forensic Science International: Genetics 65:102865. doi:10.1016/j.fsigen.2023.102865 37004371

[2] Liu G, Li T, Zhu X, Zhang X, Wang J. 2023. An independent evaluation in a CRC patient cohort of microbiome 16S rRNA sequence analysis methods: OTU clustering, DADA2, and deblur. Front Microbiol 14:1178744. doi:10.3389/fmicb.2023.1178744 37560524 PMC10408458

[3] Bokulich NA, Łaniewski P, Adamov A, Chase DM, Caporaso JG, Herbst-Kralovetz MM. 2022. Multi-omics data integration reveals metabolome as the top predictor of the cervicovaginal microenvironment. PLoS Comput Biol 18:e1009876. doi:10.1371/journal.pcbi.1009876 35196323 PMC8901057

[4] Costello EK, Lauber CL, Hamady M, Fierer N, Gordon JI, Knight R. 2009. Bacterial community variation in human body habitats across space and time. Science 326:1694–1697. doi:10.1126/science.1177486 19892944 PMC3602444

[5] Franzosa EA, Huang K, Meadow JF, Gevers D, Lemon KP, Bohannan BJM, Huttenhower C. 2015. Identifying personal microbiomes using metagenomic codes. Proc Natl Acad Sci USA 112:E2930–8. doi:10.1073/pnas.1423854112 25964341 PMC4460507

[6] Díez López C, Montiel González D, Haas C, Vidaki A, Kayser M. 2020. Microbiome-based body site of origin classification of forensically relevant blood traces. Forensic Sci Int Genet 47:102280. doi:10.1016/j.fsigen.2020.102280 32244163

[7] Schmedes SE, Woerner AE, Novroski NMM, Wendt FR, King JL, Stephens KM, Budowle B. 2018. Targeted sequencing of clade-specific markers from skin microbiomes for forensic human identification. Forensic Sci Int Genet 32:50–61. doi:10.1016/j.fsigen.2017.10.004 29065388

[8] Woerner AE, Novroski NMM, Wendt FR, Ambers A, Wiley R, Schmedes SE, Budowle B. 2019. Forensic human identification with targeted microbiome markers using nearest neighbor classification. Forensic Sci Int Genet 38:130–139. doi:10.1016/j.fsigen.2018.10.003 30396009

[9] Hanssen EN, Avershina E, Rudi K, Gill P, Snipen L. 2017. Body fluid prediction from microbial patterns for forensic application. Forensic Sci Int Genet 30:10–17. doi:10.1016/j.fsigen.2017.05.009 28605650

[10] Díez López C, Vidaki A, Ralf A, Montiel González D, Radjabzadeh D, Kraaij R, Uitterlinden AG, Haas C, Lao O, Kayser M. 2019. Novel taxonomy-independent deep learning microbiome approach allows for accurate classification of different forensically relevant human epithelial materials. Forensic Sci Int Genet 41:72–82. doi:10.1016/j.fsigen.2019.03.015 31003081

[11] Dobay A, Haas C, Fucile G, Downey N, Morrison HG, Kratzer A, Arora N. 2019. Microbiome-based body fluid identification of samples exposed to indoor conditions. Forensic Sci Int Genet 40:105–113. doi:10.1016/j.fsigen.2019.02.010 30785061

[12] Metcalf JL, Xu ZZ, Bouslimani A, Dorrestein P, Carter DO, Knight R. 2017. Microbiome tools for forensic science. Trends Biotechnol 35:814–823. doi:10.1016/j.tibtech.2017.03.006 28366290

[13] Swayambhu M, Kümmerli R, Arora N. 2023. Microbiome-based stain analyses in crime scenes. Appl Environ Microbiol 89:e0132522. doi:10.1128/aem.01325-22 36625592 PMC9888269

[14] Abellan-Schneyder I, Matchado MS, Reitmeier S, Sommer A, Sewald Z, Baumbach J, List M, Neuhaus K. 2021. Primer, pipelines, parameters: issues in 16S rRNA gene sequencing. mSphere 6. doi:10.1128/mSphere.01202-20 PMC8544895 33627512

[15] Jones CB, White JR, Ernst SE, Sfanos KS, Peiffer LB. 2022. Incorporation of data from multiple hypervariable regions when analyzing bacterial 16S rRNA gene sequencing data. Front Genet 13:799615. doi:10.3389/fgene.2022.799615 35432480 PMC9009396

[16] Tackmann J, Arora N, Schmidt TSB, Rodrigues JFM, von Mering C. 2018. Ecologically informed microbial biomarkers and accurate classification of mixed and unmixed samples in an extensive cross-study of human body sites. Microbiome 6:192. doi:10.1186/s40168-018-0565-6 30355348 PMC6201589

[17] Gill P, Hicks T, Butler JM, Connolly E, Gusmão L, Kokshoorn B, Morling N, van Oorschot RAH, Parson W, Prinz M, Schneider PM, Sijen T, Taylor D. 2018. DNA commission of the international society for forensic genetics: assessing the value of forensic biological evidence - guidelines highlighting the importance of propositions: part I: evaluation of DNA profiling comparisons given (sub-) source propositions. Forensic Sci Int Genet 36:189–202. doi:10.1016/j.fsigen.2018.07.003 30041098

[18] Clark C, Turiello R, Cotton R, Landers JP. 2021. Analytical approaches to differential extraction for sexual assault evidence. Anal Chim Acta 1141:230–245. doi:10.1016/j.aca.2020.07.059 33248657

[19] Benschop CCG, Quaak FCA, Boon ME, Sijen T, Kuiper I. 2012. Vaginal microbial flora analysis by next generation sequencing and microarrays; can microbes indicate vaginal origin in a forensic context? Int J Legal Med 126:303–310. doi:10.1007/s00414-011-0660-8 22282153

[20] Vuichard S, Borer U, Bottinelli M, Cossu C, Malik N, Meier V, Gehrig C, Sulzer A, Morerod M-L, Castella V. 2011. Differential DNA extraction of challenging simulated sexual-assault samples: A Swiss collaborative study. Investig Genet 2:11. doi:10.1186/2041-2223-2-11 PMC3119174 21542912

[21] Flanagan L, Murphy C, Savage P, Breathnach M, Ryan J. 2024. The importance of male underwear in cases of alleged sexual assault. J Forensic Sci 69:1481–1489. doi:10.1111/1556-4029.15539 38703136

[22] Lebeer S, Ahannach S, Gehrmann T, Wittouck S, Eilers T, Oerlemans E, Condori S, Dillen J, Spacova I, Vander Donck L, Masquillier C, Allonsius CN, Bron PA, Van Beeck W, De Backer C, Donders G, Verhoeven V. 2023. A citizen-science-enabled catalogue of the vaginal microbiome and associated factors. Nat Microbiol 8:2183–2195. doi:10.1038/s41564-023-01500-0 37884815 PMC10627828

[23] Meisel JS, Hannigan GD, Tyldsley AS, SanMiguel AJ, Hodkinson BP, Zheng Q, Grice EA. 2016. Skin microbiome surveys are strongly influenced by experimental design. J Invest Dermatol 136:947–956. doi:10.1016/j.jid.2016.01.016 26829039 PMC4842136

[24] Seashols-Williams S, Green R, Wohlfahrt D, Brand A, Tan-Torres AL, Nogales F, Brooks JP, Singh B. 2018. An accurate bacterial DNA quantification assay for HTS library preparation of human biological samples. Electrophoresis 39:2824–2832. doi:10.1002/elps.201800127 29772600

[25] Cauwenberghs E, Oerlemans E, Wittouck S, Allonsius CN, Gehrmann T, Ahannach S, De Boeck I, Spacova I, Bron PA, Donders G, Verhoeven V, Lebeer S. 2023. Salivary microbiome of healthy women of reproductive age. MBio 14:e0030023. doi:10.1128/mbio.00300-23 37655878 PMC10653790

[26] Chiarello M, McCauley M, Villéger S, Jackson CR. 2021. Ranking the biases: the choice of OTUs vs. ASVs in 16S rRNA amplicon data analysis has stronger effects on diversity measures than rarefaction and similarity threshold. Internet. Internet, In Review. doi:10.21203/rs.3.rs-764430/v1 PMC8870492 35202411

[27] Hakimzadeh A, Abdala Asbun A, Albanese D, Bernard M, Buchner D, Callahan B, Caporaso JG, Curd E, Djemiel C, Brandström Durling M, et al.. 2024. A pile of pipelines: an overview of the bioinformatics software for metabarcoding data analyses. Mol Ecol Resour 24:e13847. doi:10.1111/1755-0998.13847 37548515 PMC10847385

[28] Maruyama H, Masago A, Nambu T, Mashimo C, Okinaga T. 2020. Amplicon sequence variant-based oral microbiome analysis using QIIME 2. Life Sci. doi:10.20944/preprints202008.0206.v1

[29] Reitmeier S, Hitch TCA, Treichel N, Fikas N, Hausmann B, Ramer-Tait AE, Neuhaus K, Berry D, Haller D, Lagkouvardos I, Clavel T. 2021. Handling of spurious sequences affects the outcome of high-throughput 16S rRNA gene amplicon profiling. ISME Commun 1:31. doi:10.1038/s43705-021-00033-z 37938227 PMC9723555

[30] Schloss PD. 2021. Amplicon sequence variants artificially split bacterial genomes into separate clusters. mSphere 6:e0019121. doi:10.1128/mSphere.00191-21 34287003 PMC8386465

[31] Johnson JS, Spakowicz DJ, Hong B-Y, Petersen LM, Demkowicz P, Chen L, Leopold SR, Hanson BM, Agresta HO, Gerstein M, Sodergren E, Weinstock GM. 2019. Evaluation of 16S rRNA gene sequencing for species and strain-level microbiome analysis. Nat Commun 10:5029. doi:10.1038/s41467-019-13036-1 31695033 PMC6834636

[32] Carter KA, France MT, Rutt L, Bilski L, Martinez-Greiwe S, Regan M, Brotman RM, Ravel J. 2024. Sexual transmission of urogenital bacteria: whole metagenome sequencing evidence from a sexual network study. mSphere 9:e0003024. doi:10.1128/msphere.00030-24 38358269 PMC10964427

[33] Alam MT, Amos GCA, Murphy ARJ, Murch S, Wellington EMH, Arasaradnam RP. 2020. Microbial imbalance in inflammatory bowel disease patients at different taxonomic levels. Gut Pathog 12. doi:10.1186/s13099-019-0341-6 PMC6942256 31911822

[34] Thompson WC, Taroni F, Aitken CGG. 2003. How the probability of a false positive affects the value of DNA evidence. J Forensic Sci 48:47–54. 12570198

[35] Asnicar F, Thomas AM, Passerini A, Waldron L, Segata N. 2024. Machine learning for microbiologists. Nat Rev Microbiol 22:191–205. doi:10.1038/s41579-023-00984-1 37968359 PMC11980903

[36] Samie L, Champod C, Delémont S, Basset P, Hicks T, Castella V. 2022. Use of Bayesian Networks for the investigation of the nature of biological material in casework. Forensic Sci Int 331:111174. doi:10.1016/j.forsciint.2022.111174 34999364

[37] Noyes N, Cho KC, Ravel J, Forney LJ, Abdo Z. 2018. Associations between sexual habits, menstrual hygiene practices, demographics and the vaginal microbiome as revealed by Bayesian network analysis. PLoS One 13:e0191625. doi:10.1371/journal.pone.0191625 29364944 PMC5783405

[38] Ahannach S, Gehrmann T, Spacova I, Wittouck S, Hiers J, Bron P, Donck LV, Cromphout M, Swayambhu M, Arora N, Schuh L, Tournoy I, Smeers I, Wuestenbergs J, Bekaert B, Decorte R, Jehaes E, Lebeer S. 2024. Microbial and seminal traces of sexual intercourse and forensic implications. In Review. doi:10.21203/rs.3.rs-4302243/v1 PMC12632010 41267120

[39] Carda-Diéguez M, Cárdenas N, Aparicio M, Beltrán D, Rodríguez JM, Mira A. 2019. Variations in vaginal, penile, and oral microbiota after sexual intercourse: a case report. Front Med (Lausanne) 6:178. doi:10.3389/fmed.2019.00178 31440511 PMC6692966

[40] Dixon R, Egan S, Hughes S, Chapman B. 2023. The sexome ‐ a proof of concept study into microbial transfer between heterosexual couples after sexual intercourse. Forensic Sci Int 348:111711. doi:10.1016/j.forsciint.2023.111711 37224760

[41] Zozaya M, Ferris MJ, Siren JD, Lillis R, Myers L, Nsuami MJ, Eren AM, Brown J, Taylor CM, Martin DH. 2016. Bacterial communities in penile skin, male urethra, and vaginas of heterosexual couples with and without bacterial vaginosis. Microbiome 4:16. doi:10.1186/s40168-016-0161-6 27090518 PMC4835890

[42] Mändar R, Punab M, Borovkova N, Lapp E, Kiiker R, Korrovits P, Metspalu A, Krjutškov K, Nõlvak H, Preem J-K, Oopkaup K, Salumets A, Truu J. 2015. Complementary seminovaginal microbiome in couples. Res Microbiol 166:440–447. doi:10.1016/j.resmic.2015.03.009 25869222

[43] Harbison S, Fleming R. 2016. Forensic body fluid identification: state of the art. RRFMS:11. doi:10.2147/RRFMS.S57994

[44] Sijen T. 2015. Molecular approaches for forensic cell type identification: On mRNA, miRNA, DNA methylation and microbial markers. Forensic Sci Int Genet 18:21–32. doi:10.1016/j.fsigen.2014.11.015 25488609

[45] Parker GJ, McKiernan HE, Legg KM, Goecker ZC. 2021. Forensic proteomics. Forensic Sci Int Genet 54:102529. doi:10.1016/j.fsigen.2021.102529 34139528

[46] Ingold S, Dørum G, Hanson E, Ballard D, Berti A, Gettings KB, Giangasparo F, Kampmann M-L, Laurent F-X, Morling N, Parson W, Steffen CR, Ulus A, van den Berge M, van der Gaag KJ, Verdoliva V, Xavier C, Ballantyne J, Haas C. 2020. Body fluid identification and assignment to donors using a targeted mRNA massively parallel sequencing approach - results of a second EUROFORGEN/EDNAP collaborative exercise. Forensic Sci Int Genet 45:102208. doi:10.1016/j.fsigen.2019.102208 31869731

[47] Neis M, Groß T, Schneider H, Schneider PM, Courts C. 2024. Comprehensive body fluid identification and contributor assignment by combining targeted sequencing of mRNA and coding region SNPs. Forensic Sci Int Genet 73:103125. doi:10.1016/j.fsigen.2024.103125 39182373

[48] Park J-L, Kwon O-H, Kim JH, Yoo H-S, Lee H-C, Woo K-M, Kim S-Y, Lee S-H, Kim YS. 2014. Identification of body fluid-specific DNA methylation markers for use in forensic science. Forensic Sci Int Genet 13:147–153. doi:10.1016/j.fsigen.2014.07.011 25128690

[49] Kamanna S, Henry J, Voelcker NH, Linacre A, Kirkbride KP. 2016. Direct identification of forensic body fluids using matrix-assisted laser desorption/ionization time-of-flight mass spectrometry. Int J Mass Spectrom 397–398:18–26. doi:10.1016/j.ijms.2016.01.002

[50] Kusano M, Mendez E, Furton KG. 2013. Comparison of the volatile organic compounds from different biological specimens for profiling potential. J Forensic Sci 58:29–39. doi:10.1111/j.1556-4029.2012.02215.x 22803833

[51] Rankin-Turner S, Turner MA, Kelly PF, King RSP, Reynolds JC. 2019. Transforming presumptive forensic testing: in situ identification and age estimation of human bodily fluids. Chem Sci 10:1064–1069. doi:10.1039/C8SC04133D 30774902 PMC6346410

[52] Yang H, Zhou B, Deng H, Prinz M, Siegel D. 2013. Body fluid identification by mass spectrometry. Int J Legal Med 127:1065–1077. doi:10.1007/s00414-013-0848-1 23525663

[53] Poggiali B, Dupont ME, Jacobsen SB, Smerup MH, Christiansen SNN, Tfelt-Hansen J, Vidaki A, Morling N, Andersen JD. 2024. DNA methylation stability in cardiac tissues kept at different temperatures and time intervals. Sci Rep 14:25170. doi:10.1038/s41598-024-76027-3 39448773 PMC11502879

[54] Kohlmeier F, Schneider PM. 2012. Successful mRNA profiling of 23 years old blood stains. Forensic Sci Int Genet 6:274–276. doi:10.1016/j.fsigen.2011.04.007 21612994

[55] UC DAVIS DNA Technologies Core. 2024 How do I size select libraries for the HiSeq 4000 with beads. https://dnatech.ucdavis.edu/faqs/how-do-i-size-select-libraries-for-the-hiseq-4000-with-beads/?

[56] Callahan BJ, McMurdie PJ, Rosen MJ, Han AW, Johnson AJA, Holmes SP. 2016. DADA2: high-resolution sample inference from Illumina amplicon data. Nat Methods 13:581–583. doi:10.1038/nmeth.3869 27214047 PMC4927377

[57] Pruesse E, Quast C, Knittel K, Fuchs BM, Ludwig W, Peplies J, Glöckner FO. 2007. SILVA: a comprehensive online resource for quality checked and aligned ribosomal RNA sequence data compatible with ARB. Nucleic Acids Res 35:7188–7196. doi:10.1093/nar/gkm864 17947321 PMC2175337

[58] Robeson MS 2nd, O’Rourke DR, Kaehler BD, Ziemski M, Dillon MR, Foster JT, Bokulich NA. 2021. RESCRIPt: reproducible sequence taxonomy reference database management. PLoS Comput Biol 17:e1009581. doi:10.1371/journal.pcbi.1009581 34748542 PMC8601625

[59] Quast C, Pruesse E, Yilmaz P, Gerken J, Schweer T, Yarza P, Peplies J, Glöckner FO. 2013. The SILVA ribosomal RNA gene database project: improved data processing and web-based tools. Nucleic Acids Res 41:D590–6. doi:10.1093/nar/gks1219 23193283 PMC3531112

[60] Bolyen E, Rideout JR, Dillon MR, Bokulich NA, Abnet CC, Al-Ghalith GA, Alexander H, Alm EJ, Arumugam M, Asnicar F, et al.. 2019. Reproducible, interactive, scalable and extensible microbiome data science using QIIME 2. Nat Biotechnol 37:852–857. doi:10.1038/s41587-019-0209-9 31341288 PMC7015180

[61] Davis NM, Proctor DM, Holmes SP, Relman DA, Callahan BJ. 2018. Simple statistical identification and removal of contaminant sequences in marker-gene and metagenomics data. Microbiome 6:226. doi:10.1186/s40168-018-0605-2 30558668 PMC6298009

[62] Bokulich NA, Dillon MR, Bolyen E, Kaehler BD, Huttley GA, Caporaso JG. 2018. Q2-sample-classifier: machine-learning tools for microbiome classification and regression. J Open Res Softw 3:934. doi:10.21105/joss.00934 31552137 PMC6759219

[63] Pedregosa F, Varoquaux G, Gramfort A, Michel V, Thirion B, Grisel O, Blondel M, Prettenhofer P, Weiss R, Dubourg V, Vanderplas J, Passos A, Cournapeau D, Brucher M, Perrot M, Duchesnay É. 2011. Scikit-learn: machine learning in python. J Mach Learn Res 12:2825–2830.

