# Supplementary material for: Standardizing a microbiome pipeline for body fluid identification from complex crime scene stains
Source: Appl Environ Microbiol. 2025 Apr 30;91(5):e01871-24. doi: 10.1128/aem.01871-24 (PMC12093949; doi:10.1128/aem.01871-24)
Supplement: File S1 — Additional PCoA plots, regression analyses, performance metrics of the classifiers, and sample design for the mock data sets. [file aem.01871-24-s0001.pdf]

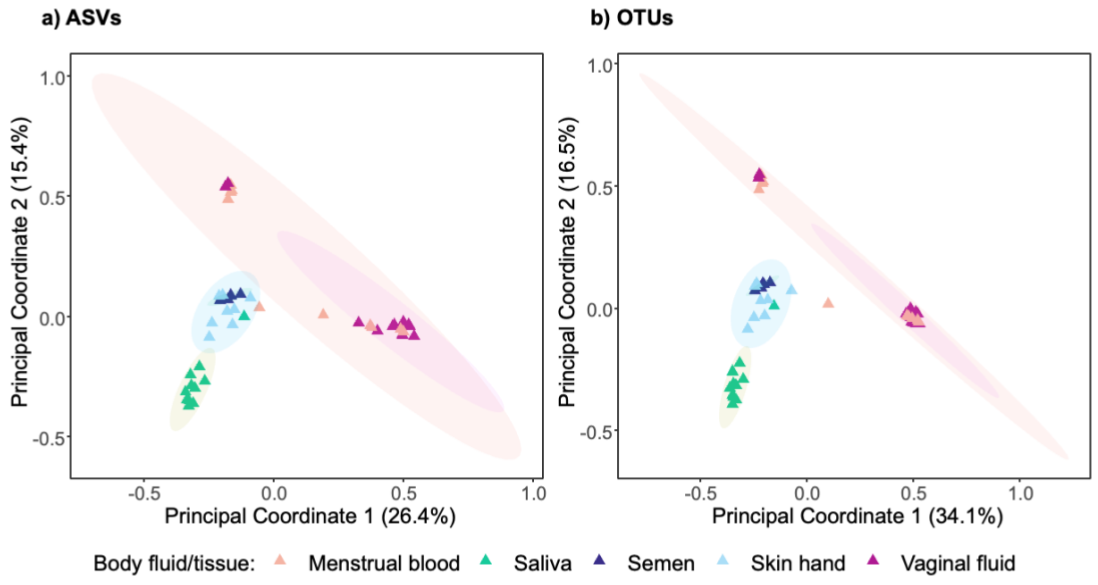

Figure S1.1 Principal coordinate analysis plots (based on the Bray Curtis distances) showing clustering of body fluid/tissue samples. a) *ASV data for samples from Dobay et al. (n=42, PERMANOVA $F_{4,41}$ = 6.15, r^2 = 0.38, p = 0.001),* ***b)*** *OTU data clustered at 97% for samples from Dobay et al. (n=42,* PERMANOVA $F_{4,41}$ = 7.64, r^2 = 0.43, p = 0.001 *). Body fluid/tissues are colour coded.*

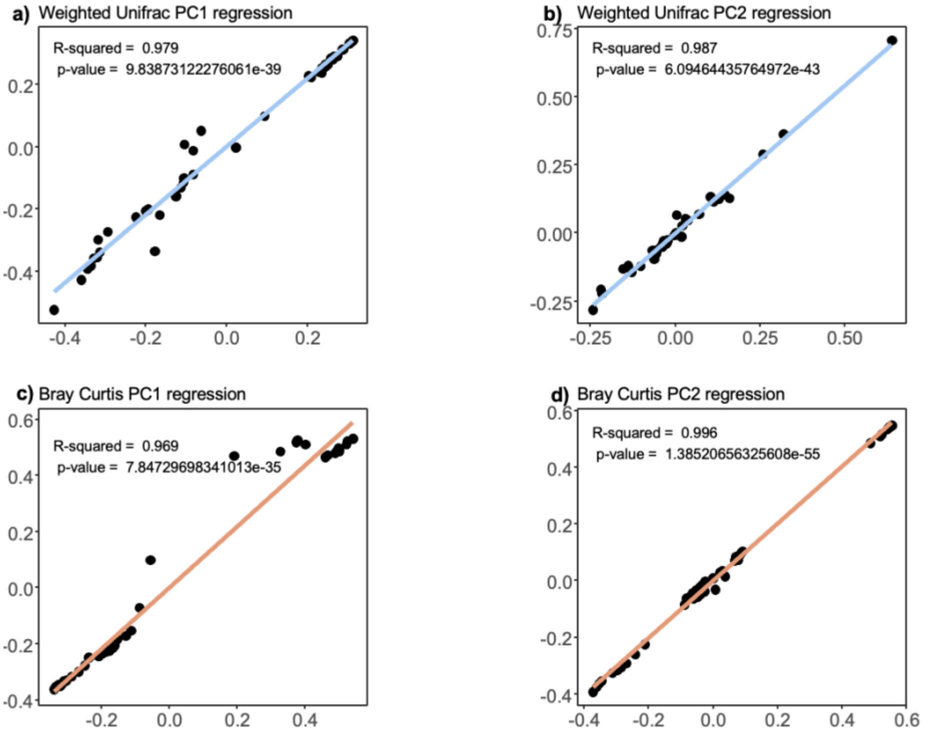

Figure S1.2 Regression analyses for PC1 and PC2 coordinates for weighted Unifrac and Bray Curtis PCoA plots. a) *Weighted Unifrac PC1 regression,* ***b)****Weighted Unifrac PC2 regression,* ***c)*** *Bray Curtis PC1 regression,* ***d)*** *Bray Curtis PC2 regression. Regression lines are coloured according to the distances.*

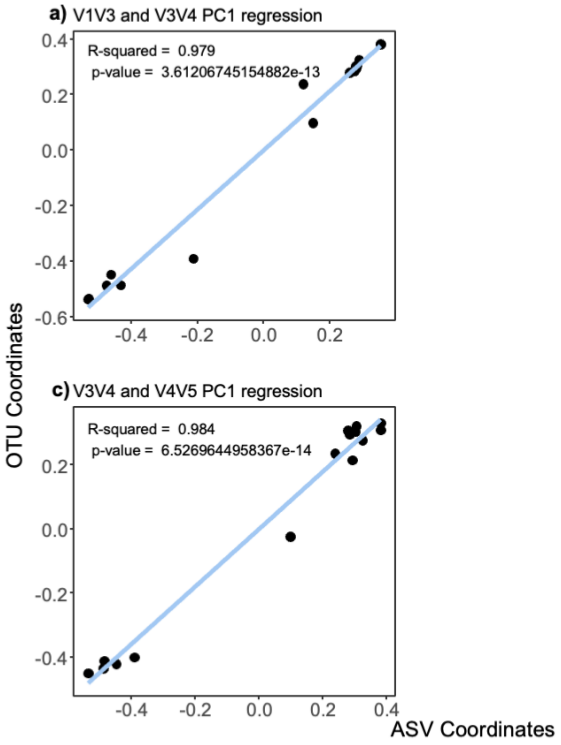

Figure S1.3 Regression analyses for PC1 and PC2 coordinates for V1V3 and V3V4; V3V4 and V4V5 coordinates. a) *V1V3 and V3V4 PC1 regression,* ***b)****V1V3 and V3V4 PC2 regression,* ***c)*** *V3V4 and V4V5 PC1 regression,* ***d)*** *V3V4 and V4V5 PC2 regression.*

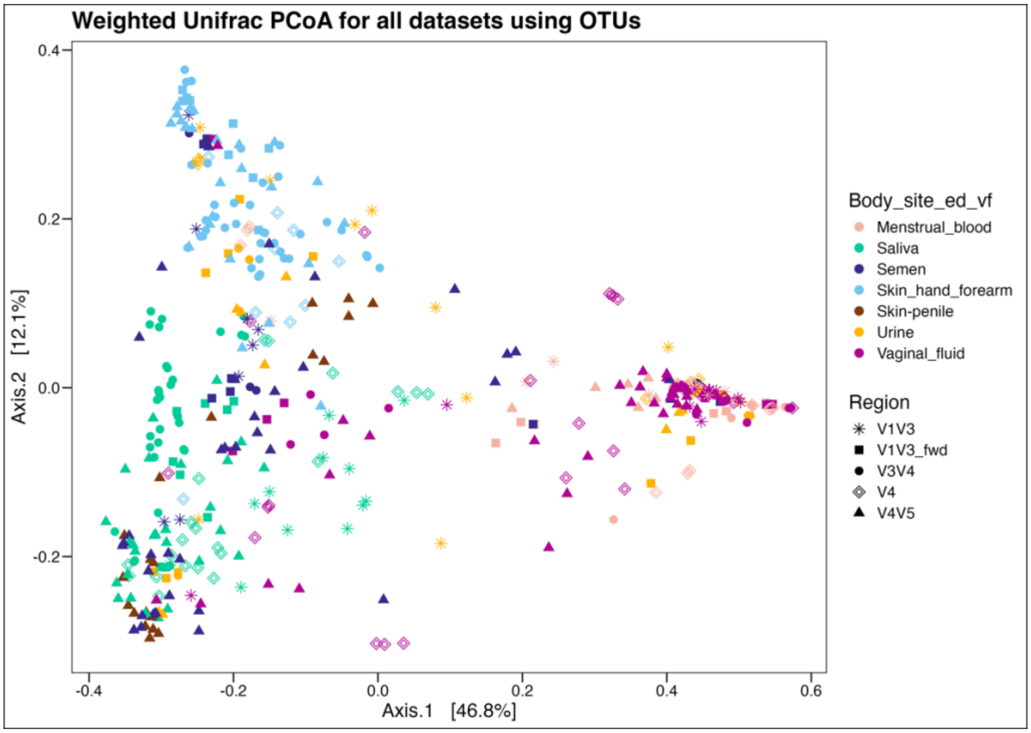

Figure S1.4 Principal coordinate analysis plots for data from V1V3, V3V4, V4 and V4V5 16S rRNA gene regions using OTUs (97%) and weighted unifrac distances for 457 samples. *(PERMANOVA with body-site $F_{6,450}$ = 55.33, r^2 = 0.42, p = 0.001, PERMANOVA with region $F_{4,452}$ = 6.82, r^2 = 0.06, p = 0.001, PERMANOVA with extraction kit: $F_{8,448}$=7.42, r^2=0.117, p=0.001)*

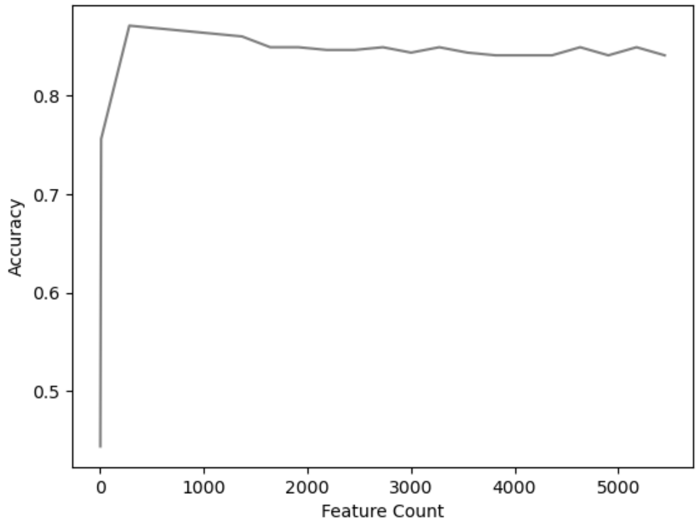

Figure S1.5 Recursive feature extraction plot for the classifier trained on 365 samples.

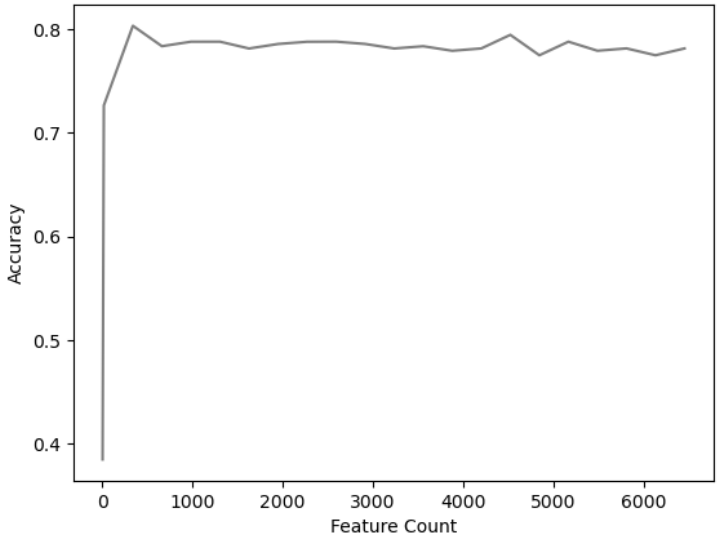

Figure S1.6 Recursive feature extraction plot for the classifier trained on 457 samples.

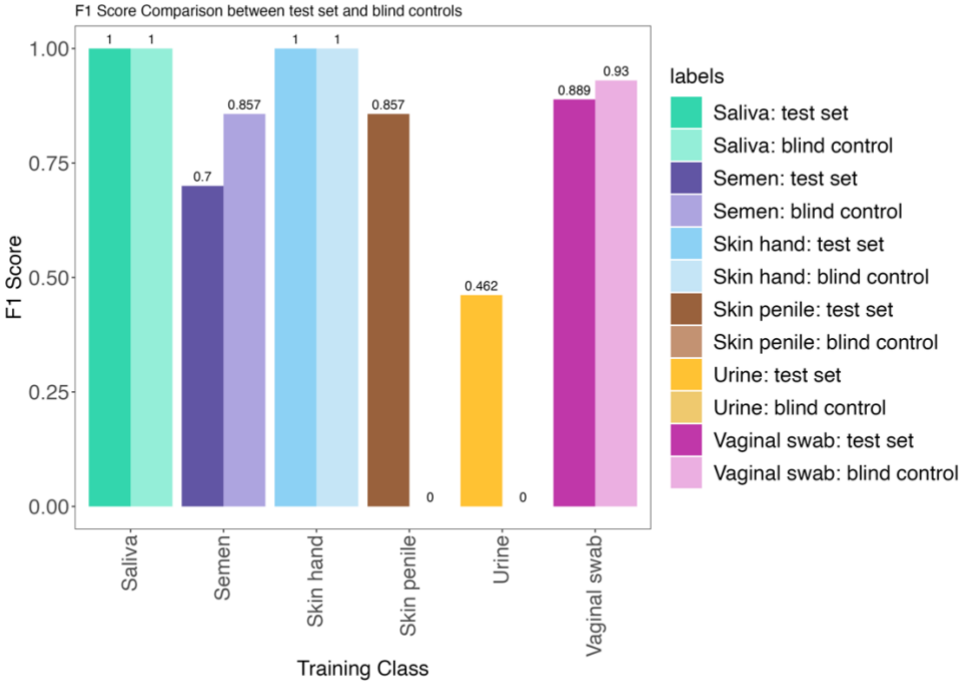

Figure S1.7 Classifier performance comparison of the classifier trained on 365 samples with test samples vs classifier trained on 457 samples with blind control samples. *Barplots depict F1 scores per class and are color coded. Vaginal swab refers to both vaginal fluid and menstrual blood.*

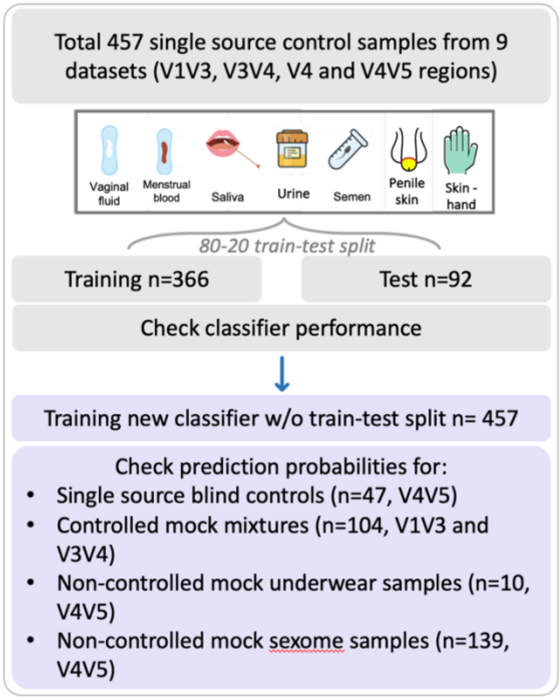

Figure S1.8 Illustrates an overview of datasets used in classifier training and testing.
