# Supplementary material for: Standardizing a microbiome pipeline for body fluid identification from complex crime scene stains
Source: Appl Environ Microbiol. 2025 Apr 30;91(5):e01871-24. doi: 10.1128/aem.01871-24 (PMC12093949; doi:10.1128/aem.01871-24)
Supplement: File S2 — Pairwise comparisons for OTU vs ASV plots. [file aem.01871-24-s0002.pdf]

OTU_weighted Unifrac

pairs	Df	SumsOfSqs	F.Model	R2	p.value	p.adjusted	sig	significant
saliva vs vaginalswab	1	2.38196371	36.5437464	0.63506026	0.001	0.01	*	Yes
saliva vs menstrualblood	1	1.76723845	21.6592731	0.53270193	0.001	0.01	*	Yes
saliva vs semen	1	0.46783231	4.16600866	0.22932988	0.007	0.07		No
saliva vs skin	1	0.7354721	6.69267628	0.2824787	0.001	0.01	*	Yes
vaginalswab vs menstrualblood	1	0.04382965	2.45607925	0.10937258	0.043	0.43		No
vaginalswab vs semen	1	1.06938664	42.345117	0.73842585	0.001	0.01	*	Yes
vaginalswab vs skin	1	1.00706704	26.8626201	0.59877511	0.001	0.01	*	Yes
menstrualblood vs semen	1	0.81037405	18.8032578	0.59123685	0.001	0.01	*	Yes
menstrualblood vs skin	1	0.66715159	12.466214	0.43793017	0.001	0.01	*	Yes
semen vs skin	1	0.25323631	3.17204278	0.22382396	0.013	0.13		No

ASV_ weighted Unifrac

pairs	Df	SumsOfSqs	F.Model	R2	p.value	p.adjusted	sig	significant
saliva vs vaginalswab	1	2.0015269	35.966094	0.63135967	0.001	0.01	*	Yes
saliva vs menstrualblood	1	1.51536982	21.4687142	0.53050152	0.001	0.01	*	Yes
saliva vs semen	1	0.42747714	4.6789433	0.25049293	0.005	0.05	.	No
saliva vs skin	1	0.60379395	6.35524005	0.27211196	0.002	0.02	.	Yes
vaginalswab vs menstrualblood	1	0.03711186	2.21916155	0.09987603	0.063	0.63		No
vaginalswab vs semen	1	0.82657314	45.513062	0.75211963	0.002	0.02	.	Yes
vaginalswab vs skin	1	0.98666688	29.1877151	0.61854479	0.001	0.01	*	Yes
menstrualblood vs semen	1	0.64077627	18.7245265	0.59022241	0.002	0.02	.	Yes
menstrualblood vs skin	1	0.68227083	13.9785818	0.46628563	0.001	0.01	*	Yes
semen vs skin	1	0.19671128	3.00997737	0.21484527	0.016	0.16		No

OTU_Bray Curtis

pairs	Df	SumsOfSqs	F.Model	R2	p.value	p.adjusted	sig	significant
saliva vs vaginalswab	1	3.59034294	19.5336548	0.481912	0.001	0.01	*	Yes
saliva vs menstrualblood	1	2.55176667	10.2746514	0.35097434	0.001	0.01	*	Yes
saliva vs semen	1	1.43726421	6.20175064	0.30699075	0.001	0.01	*	Yes
saliva vs skin	1	1.62224112	6.53693559	0.27773096	0.001	0.01	*	Yes
vaginalswab vs menstrualblood	1	0.35788203	1.64224186	0.07588132	0.222	1		No
vaginalswab vs semen	1	1.89989215	9.88079082	0.39712527	0.002	0.02	.	Yes
vaginalswab vs skin	1	2.37118473	11.0615992	0.38062596	0.001	0.01	*	Yes
menstrualblood vs semen	1	1.2708133	4.4135898	0.25345663	0.003	0.03	.	Yes
menstrualblood vs skin	1	1.50091366	5.0906395	0.24136961	0.001	0.01	*	Yes
semen vs skin	1	0.71245446	2.41646202	0.18011172	0.003	0.03	.	Yes

ASV_Bray curtis

pairs	Df	SumsOfSqs	F.Model	R2	p.value	p.adjusted	sig	significant
saliva vs vaginalswab	1	3.20865446	14.4699003	0.40794872	0.001	0.01	*	Yes
saliva vs menstrualblood	1	2.21783386	7.8099438	0.29130773	0.001	0.01	*	Yes
saliva vs semen	1	1.39691826	5.71968706	0.29004959	0.001	0.01	*	Yes
saliva vs skin	1	1.58960559	6.11751773	0.26462693	0.001	0.01	*	Yes
vaginalswab vs menstrualblood	1	0.58359114	2.10720767	0.09531768	0.096	0.96		No
vaginalswab vs semen	1	1.70223448	7.16694048	0.32331663	0.001	0.01	*	Yes
vaginalswab vs skin	1	2.15955031	8.52299825	0.3213437	0.001	0.01	*	Yes
menstrualblood vs semen	1	1.09774737	3.31758741	0.2033136	0.007	0.07		No
menstrualblood vs skin	1	1.29659763	3.91449391	0.19656507	0.001	0.01	*	Yes
semen vs skin	1	0.71311029	2.36032627	0.17666681	0.002	0.02	.	Yes
