# Supplementary material for: Standardizing a microbiome pipeline for body fluid identification from complex crime scene stains
Source: Appl Environ Microbiol. 2025 Apr 30;91(5):e01871-24. doi: 10.1128/aem.01871-24 (PMC12093949; doi:10.1128/aem.01871-24)
Supplement: File S9 — Legends for all supplemental material. [file aem.01871-24-s0009.docx]

Supplementary S1: Additional PCoA plots, regression analyses, performance metrics of the classifiers and sample design for the mock datasets.

Supplementary S2: Pairwise comparisons for OTUs vs ASVs plots based on weighted Unifrac and Bray curtis distances.

Supplementary S3: Additional details for laboratory and computational methods.

Supplementary S4: Prediction probabilities for the test set samples.

Supplementary S5: Prediction probabilities for the blind dataset samples.

Supplementary S6: Prediction probabilities for the mixed-source samples generated in the laboratory, sexome samples and underwear samples.

Supplementary S7: Results for ANOVA with Tukey post-hoc analysisfor mixed-source samples generated in the laboratory.

Supplementary S8: Metadata files for training and mock datasets used in the study.

Supplementary S9: Supplementary material legends.
